# DE-CMR and MPS for assessment of myocardial viability: to what extent do the two techniques agree?

**DOI:** 10.1186/1532-429X-18-S1-P88

**Published:** 2016-01-27

**Authors:** Thomas Alway, Charles Butcher, Evangelos Skondros, Richard Underwood, Joyce Wong

**Affiliations:** Imaging, Royal Brompton and Harefield Foundation Trust, London, UK

## Background

DE-CMR (gadolinium contrast enhanced cardiac magnetic resonance imaging) and MPS (nuclear myocardial perfusion scanning) are two widely used techniques to assess myocardial ‘viability’. However each evaluates different aspects of the myocardium where MRI examines scar burden whilst resting MPS assesses respiring myocytes. DE-CMR offers better spatial and temporal resolution, but requires breath holding. MPS artefacts can result from attenuation and tracer energy differences. Both techniques are limited by gating and partial volume. We investigated the agreement between the two modalities when assessing myocardial viability, in particular the effect of wall thickening assessed on MPS.

## Methods

Patients were retrospectively identified over a period between October 2010 and May 2013. Each patient had undergone MPS and MRI scans within 6 months, with no intervening infarction or revascularisation. Patients with poor image quality were excluded from the comparison. For the MPS scans, GTN was given in 50% of patients, and tetrofosmin was used in 48%. A 17 segment model of the LV was used by 2 independent observers in each modality to blindly score for processed counts, wall thickening and wall motion (resting MPS) or DE extent, wall thinning and wall motion (DE-CMR) on a scale of 0-4. A kappa statistic for agreement (SPSS 22) was performed, with a segmental analysis for overall agreement on assessment of processed counts and extent of DE, as well as between DE-CMR and MPS wall motion scores. 42 patients with IHD were further analysed separately and each of the above imaging variables in both techniques were compared on a segmental basis.

## Results

52 patients (41 male, age 63.2+/- 11.1 years) were included in the study. The commonest indications for imaging were assessment of viability (78%), diagnosis (11%), and risk stratification (1%). The commonest underlying diagnoses were ischaemic heart disease (81%), followed by cardiomyopathy (8%), arrhythmia (8%), and valvulopathy (4%).

Overall, Kappa statistical analysis showed limited agreement in either viability or motion between techniques (tables enclosed).

Within the cohort with established coronary disease, again limited agreement was noted between imaging variable cross modalities.

There was more tendency to agreement in assessment of viability than motion. Areas showing the most agreement were the apex, apical anterior and basal infero-lateral segments.

## Conclusions

DE-CMR and resting MPS measure different myocardial properties and segmental assessment results may vary. Further comparison of the two techniques with a larger study population and follow up data following optimal treatment could be useful.

**Table 1:** Table 1

Age (Mean ± S.D)	63.2 ± 11.1		
Male (n, %)	41 (78.8)		
Diagnosis (n, %)			
Ischaemic heart disease	42 (80.8)		
Cardiomyopathy	4 (7.69)		
Arrhythmia	4 (7.69)		
Valvular	2 (3.85)		
Co-morbidities/Risk factors (n, %)			
Diabetes	7 (13.5)		
Hypertension	29 (55.8)		
Hypercholesterolaemia	18 (34.6)		
Smoker/ex-smoker	18 (34.6)		
Previous intervention	30 (57.7)		
PCI	27 (51.9)		
CABG	2 (3.85)		
Previous MI	38 (73.1)		
Pulmonary disease	12 (23.1)		
Known CAD	43 (82.7)		
AF	6 (11.5)		
LBBB	4 (7.69)		
Outcomes (n, %)			
Alive	51 (98.1)		
Revascularised after scans	13 (25.0)		
Kappa statistics comparing imaging variables on DE-CMR and resting MPS			
Viable CMR segments	DE	Wall thinning	Wall motion
Processed counts	0.261	0.064	0.167
Thickening	0.267	0.056	0.218
Motion	0.195	0.065	0.167

**Figure 1:**
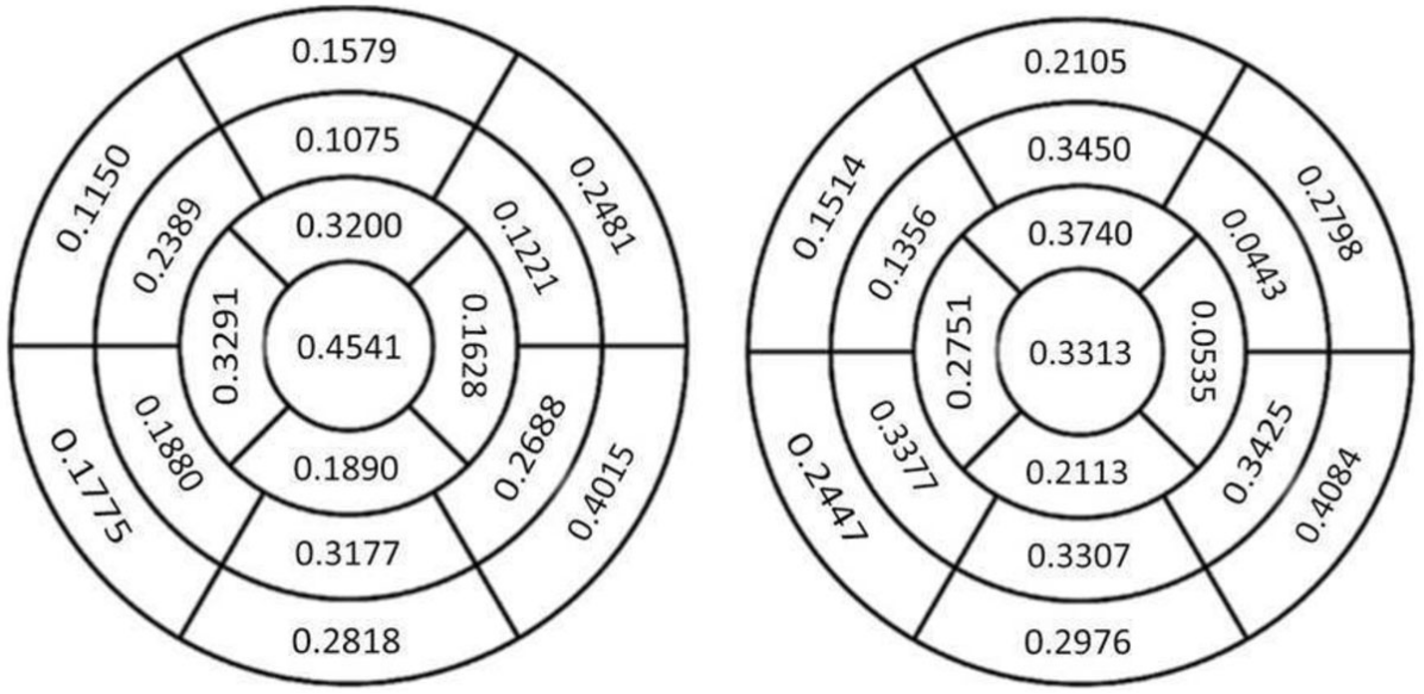
**Kappa statistics assessing segmental agreement between DE CMR and resting MPS for left ventricular wall motion and processed counts versus DE extent**.

